# Mobile cancer prevention and early detection outreach in Uganda: Partnering with communities toward bridging the cancer health disparities through “asset‐based community development model”

**DOI:** 10.1002/cam4.3387

**Published:** 2020-08-18

**Authors:** Alfred Jatho, Noleb M. Mugisha, James Kafeero, George Holoya, Fred Okuku, Nixon Niyonzima

**Affiliations:** ^1^ National Cancer Center Graduate School of Cancer Science and Policy Goyang Republic of Korea; ^2^ Uganda Cancer Institute Kampala Uganda

**Keywords:** cancer health disparity, cancer screening, community partnership, low‐income countries, mobile cancer clinic

## Abstract

**Background:**

Communities in low‐income countries are characterized by limited access to cancer prevention and early detection services, even for the commonest types of cancer. Limited resources for cancer control are one of the contributors to cancer health disparities. We explored the feasibility and benefit of conducting outreaches in partnership with local communities using the “asset‐based community development (ABCD)” model.

**Methods:**

We analyzed the quarterly Uganda cancer institute (UCI) community outreach cancer health education and screening output reported secondary data without individual identifiers from July 2016 to June 2019 to compare the UCI‐hospital‐based and community outreach cancer awareness and screening services based on the ABCD model.

**Results:**

From July 2016 to June 2019, we worked with 107 local partners and conducted 151 outreaches. Of the total number of people who attended cancer health education sessions, 201 568 (77.9%) were reached through outreaches. Ninety‐two (95%) cancer awareness TVs and radio talk‐shows conducted were sponsored by local partners. Of the total people screened; 22 795 (63.0%) cervical, 22 014 (64.4%) breast, and 4904 (38.7%) prostate screening were reached through community outreach model. The screen‐positive rates were higher in hospital‐based screening except for Prostate screening; cervical, 8.8%, breast, 8.4%, prostate, 7.1% than in outreaches; cervical, 3.2%, breast, 2.2%, prostate, 8.2%. Of the screened positive clients who were eligible for precancer treatment like cryotherapy for treatment of precervical cancer lesions, thousands‐folds monetary value and productive life saved relative to the market cost of cancer treatment and survival rate in Uganda. When the total number of clients screened for cervical, breast, and prostate cancer are subjected to the incremental cost of specific screening, a greater portion (98.7%) of the outreach cost was absorbed through community partnership.

**Conclusions:**

Outreaching and working in collaboration with communities as partners through asset‐based community development model are feasible and help in cost‐sharing and leverage for scarce resources to promote primary prevention and early detection of cancer. This could contribute to bridging the cancer health disparities in the target populations.

## Graphical abstract

Outreaching and working in collaboration with communities as partners are feasible and help in cost‐sharing and leverage for scarce resources to promote primary prevention and early detection of cancer. This contributes to bridging cancer health disparity in the target population.

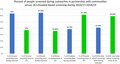

## 1 INTRODUCTION

### 1.1 Cancer health disparities

Cancer health disparity, also termed cancer health inequities refers to differences in access to cancer care, including information, early detection, treatment modalities, and cancer‐related outcomes such as incidence, prevalence, mortality, and other adverse cancer‐related health conditions among specific groups.^1^ This group differential could be based on geographical, ethnic and socioeconomic status, gender, education, culture or any other disadvantaged population groups.^1^

Limited resources for cancer control are one of the contributors of cancer health disparity. As communities in high‐income countries experience better access to cancer prevention and early detection services, and in some countries to the extent of over‐diagnosis of certain types of cancer,^2^ communities in low‐income countries are characterized by limited access to cancer prevention and early detection services, even for the commonest types of cancer. This could be attributed to the relatively higher level of cancer‐related functional health literacy, better or well‐organized cancer screening program with a fairly balanced supply of health technologies in high‐income countries compared to low‐income countries.^3^, ^4^, ^5^

The low‐income countries also experience an inadequate number of multidisciplinary cancer experts, including clinical oncologists (6). Cancer control programs are not usually the top priorities of the top‐level policymakers and international funders compared to infectious diseases in low‐income countries. The low‐income status of many African countries exacerbates this complex situation with either one or no comprehensive cancer center, opportunistic or health camp‐based screening programs. Rural and socioeconomically disadvantaged populations experience the worst difficulties in accessing cancer prevention and early detection services. Deliberate efforts are required to outreach such populations with affordable cancer preventive and early detection services.

### 1.2 Cancer early detection and treatment cost

Cancer is a costly group of diseases with complex and varying screening, diagnostic, and treatment modalities. For example, the screening, diagnostic, and treatment modalities and costs for Cervical, Breast, and Prostate cancers vary significantly even if they were of the same disease‐stage. The average cost of cervical cancer screening using Pap smear is 91Euro (US $99) in high‐income countries.^7^ The individual patient‐level clinical cost per patient including diagnostic test, staging, treatment based on the FIGO stage I‐IV cancer, chemotherapy and outpatient care increases with the stage of cancer disease. The average cost in high‐income settings varies by stages for example in Europe; 17 514 euro (18 000 USD) for FIGO Ia1‐Ib1, 43 950 for FIGO Ib2, 45 126 for FIGO II, 41 125 for FIGO III, and 51 420 for FIGO IV.^7^ This amounts to an average cost of 33 189.17 Euro, equivalent of US $36 751.07.

In East Africa, the cost of cervical cancer management in publicly funded cancer hospitals also vary by disease stage, but much lower than the cost in high‐income countries. For instance, excluding overhead cost; in Tanzania, the average hospital‐based screening cost based on visual inspection with acetic acid (VIA) per patient is US$1.45, the average cost of cryotherapy for treating cervical‐precancer lesions per patient is US$28.97 whereas the average cost of treating an early stage (stages 1 and 2) patient is US $3000.^8^ In Ghana,^9^ the incremental economic costs per client screened with VIA varied from 4.93 US$ to 14.75 US$, whereas the cost of cryotherapy varied from 47.26 US$ to 84.48 US$ whereas in base‐case assumptions modeling, the costs of VIA was found to be 6.12 US$ per woman and cost of cryotherapy was found to be 27.96 US$.

In the Medicare scheme in the United States, it is reported that the age‐standardized breast screening‐related cost per woman varied across regions from $42 to $107.^10^ The average market cost of early‐disease breast cancer surgery; lumpectomy or mastectomy in Uganda as at 2018 was 10 500 000 Uganda shillings (US$ 3000). A systematic review on global treatment costs of breast cancer on FIGO staging system,^11^ the average cumulative treatment costs weighted by sample sizes were $29 724 at stage I, $39 322 at stage II, $57 827 at stage III, and $62 108 at stage IV in 2015 US dollars. On average, costs at stage II, III, and IV were found to be 32%, 95%, and 109% higher than treatment costs at stage I. In other studies, in which invasive breast cancer was categorized as local, regional, and distant, the average weighted costs were $63 664, $89 898, and $168 906. Treatment costs of regional and distant breast cancer were found to be 41% and 165% higher than localized breast cancer on average.^11^

In a study by Fourcade et al,^12^ Prostate cancer treatment cost per patient for localized disease excluding follow‐up and adverse event cost varies by countries; 5851 Euro per patient in France, 3698 Euro per patient in Germany, 3682 Euro per patient in UK, and 10, 296 Euro in Canada, an average of 5881 Euro (6369 US$) per patient. Patients with regional prostate cancer experience higher total cost per patient to the average tune of 16, 608 euro, an equivalent of 18 000 US$.^13^, ^14^ Prostate cancer surgery on average cost 10 000 US$ per patient (Pate et al 2013). The average market cost of early‐prostate disease surgery in Uganda was 15 000 000 Uganda shillings (US $ 4286) in 2018. The average market cost of prostate screening using PSA, DRE with or without ultrasound scan in Uganda as in 2018 was estimated at 105 000 Uganda shillings (US$ 30).

### 1.3 Cancer control and the concept of mobile cancer prevention and early detection clinic in Uganda

Since 1967, Uganda has one comprehensive cancer treatment centre, the UCI, located in the Central region of Uganda, within the Capital city, Kampala. However, there is a plan for the establishment of four regional cancer centers in Western, North‐Western (West‐Nile), Northern, and Eastern Uganda. The integration of cancer information, screening and referral of suspected cancer cases in primary health‐care facilities was initiated in 2017 through training of primary health‐care workers. This is envisaged to contribute to increasing population cancer awareness to promote prevention and early detection.

In 2009 the concept of community cancer program came to reality when UCI received a donation of a 35‐foot mobile mammography unit van from Yale University/Johnson & Johnson program. The purpose of this mammography‐van that was one of the Yale‐New Haven Hospital's mammography vans was for community breast cancer education and screening services. This was Under the auspices of Yale University School of Medicine's Johnson & Johnson supported “Health Overseas Partnerships in Health and Education” (HOPE) program of 2008.

In 2015, with additional staff, the UCI‐Community cancer unit, established daily cancer information and early detection services at UCI and routine community cancer outreaches. In 2018, UCI received another mammography‐van donated by the Honorary Consul of Uganda in Mumbai, India, Madhusudan Agrawal, the Samta Foundation and Tata Group. The new mobile van is equipped with a mammography unit for breast cancer screening and space for cervical cancer screening.

The Community Program also called the Comprehensive Community Cancer Program (CCCP) is a community health section of the UCI that takes lead in the primary prevention of cancer and early detection. The goal of CCCP is to reduce cancer risk by increasing access to and utilization of cancer prevention services. This is done through mass media cancer awareness, outreach and hospital‐based health education on cancer risk factors, prevention, early detection measures, and screening for the leading cancers; cervical, breast, and prostate cancer. However, with inadequate program funding, it is unlikely to increase access to primary prevention and early detection of cancer if the community resources are not tapped to add on the allocated Government funding.

In outreach model, the minimum staffing is composed of a team of at least six staff; two nurses, one doctor or a gynecologist, one health educator, one counsellor and a driver is required to provide quality cervical screening services to an average of 32 women per day. If Breast screening is added, then, two radiographers, one biomedical technician, and one driver are added on the staff list. Where Prostate screening is on the agenda, then at least one lab technician and one additional doctor (Medical officer) is added on the list. This number is prorated based on the number, gender, and age group of people expected to turn up for the services. This adds an additional cost of transport and staff facilitation of about 700 000 Ugandan shillings (US $200), spread over 32 screening clients is an average of US$ 6.25 as the unit screening cost. When the costing criteria used in Tanzania by Nelson et al,^8^ then the unit cost for cervical screening using Visual inspection with acetic acid (VIA) as an example is US$ 1.45 plus the staff facilitation cost of US$ 6.25 totals to 7.7 US$ per screening client.

In this report, despite limited funding for primary prevention and early detection of cancer in Uganda, we share how working with local communities as partners to leverage resources for increasing access to primary prevention and early detection of cancer in Uganda is feasible. Community organizations and partnerships are pivotal components of community empowerment continuum.^15^ The ability of the community to mobilize resources both from its assets (within) and externally from beyond itself is an important factor in health promotion efforts.^16^ This is a translation of the Asset‐based community development model (ABCD) developed by John McKnight and John Kretzmann,^17^ used to discover a community's capacities and assets and to mobilize those assets for community health improvement. This focuses on the strengths of a specific community and figuring how to bring those strengths to bear for the benefit of the community.

### 1.4 Rationale for asset‐based community development (ABCD) model

The “needs‐based” or problem‐based approach to community health development is well‐intentioned by the central and local governments, nongovernmental organizations (NGOs), donors, and other actors but reflects a one‐sided deficiency view of the community profile and offers short‐term benefits.^18^ This creates a situation that render the provision of community health services like cancer prevention and early detection efforts costly to implement and denies access to services, especially for the rural, hard‐to‐reach, and hard‐to‐live populations. This is because every item is costed to be financed by the governments, NGOs, or donor agencies with other competing priorities. However, the ABCD model is an assets‐based model of community engagement and development including programming of health services that recognize the community's capacities and resources to leverage the externally‐coordinated services. This could be applied to community health services such as cancer control efforts, in particular cancer prevention and early detection.

**FIGURE 1:**
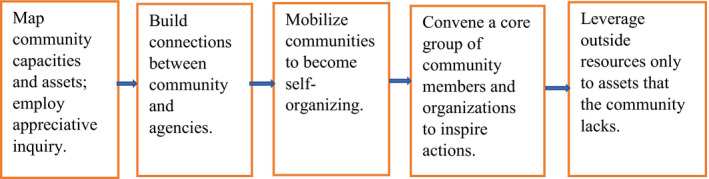
The five key stages of “assets‐based community development (ABCD) model adapted from Foot & Hopkins 2010

According to Kretzmann & McKnight “communities are built from the inside out and not from the outside in”.^19^ The ABCD model aims to identify and mobilize resources in the communities; the local governments, community‐based organizations, voluntary associations, businesses, and individuals.^20^ Foot & Hopkins^21^ outlined the key stages of ABCD model as shown in Figure 1. However, precaution must be taken not to use or view the ABCD model as means to reduce public funding for community health services nor to justify the reduced role of the state and the state related actors in providing health services for its population.^22^, ^23^, ^24^

The level of cancer awareness and access to early detection strategies such as cancer screening and early diagnosis are reported to be low in Uganda, especially in rural areas.^25^, ^26^ The capacity of the primary health care facilities to provide cancer screening in the communities is still inadequate. The UCI funding for community cancer program has been low. Up to 2015, the UCI’s (the only comprehensive cancer center in Uganda with the mandate to lead national cancer control program) approved annual government budget ceiling provided for only four long‐distance (regional/rural areas) and eight short distance cancer awareness and screening outreaches for the entire country per year and only a weekly (every Friday) screening at the Institute. During 2016‐2019, the annual budget for community outreach tripled to cover 8 long distance and 24 short distance community outreaches and daily cancer health education, information, and screening services at UCI. From the UCI’s annual work plan from 2016 to 2019, this represents an annualized average budget of 188 775 000 Uganda shillings (54 000 USD) per year. This amount excludes the African development bank funding that was prioritized for development of cancer information materials for communities and health workers and training of district PHC workers. This funding trend remains inadequate for community cancer prevention and early detection services nationally. In this situation of limited funding there is need to work with the communities as partners to leverage resources for increasing access to primary prevention and early detection of cancer in Uganda.

**FIGURE 2:**
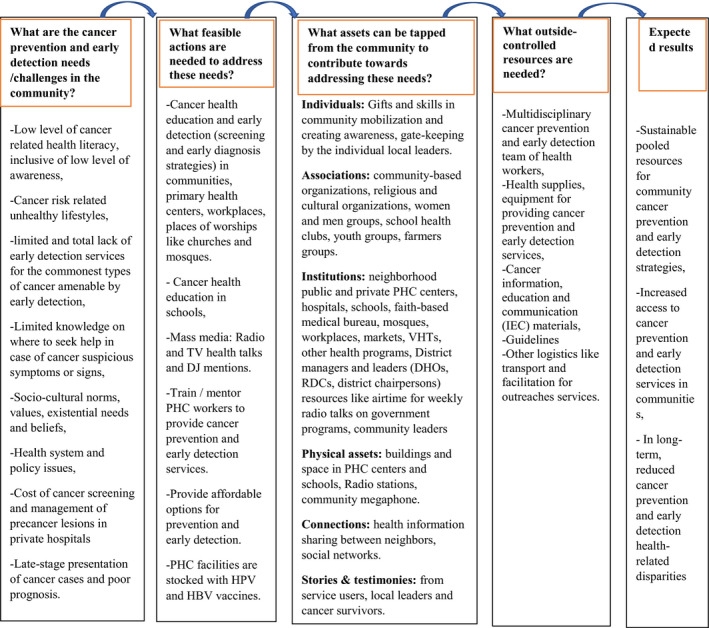
Conceptualization of ABCD model in community cancer program based on the current settings in Uganda. VHTs, village health teams (community health volunteers); PHC, primary health care; DHOs, district health officers; RDCs, resident district commissioners

Meanwhile, in the ABCD model, assets can be classified into six categories; individual, association, institutional, physical, connection and stories related community assets.^27^ We conceptualized the ABCD model to community cancer program based on the current settings in Uganda as illustrated in Figure 2. This is because the application of the ABCD model vary by context, as noted by Wildman et al “what works here does not work there.^28^” In doing this, the following were some of the questions reflected on; 1‐what are the cancer prevention and early detection needs in the communities? (sources of data: previous studies and surveys); 2‐what feasible actions are needed to address those cancer prevention and early detection related needs in the communities? (sources of data: recommendations from previous studies, surveys, knowledge of the health system and policy, evidence of what works in related settings and recommendations from international health bodies like WHO); 3‐what assets can be tapped from the communities to leverage cancer prevention and early detection? (sources of data: previous studies, surveys, knowledge of the health system, policy and sociocultural norms and values); 4‐what outside‐controlled resources are needed to provide access to cancer prevention and early detection services in the community? (Sources of data: health system, policies, guidelines, and recommendations).

Therefore, in long‐term, the implementation of this approach is anticipated to contribute in reducing the extent of the would‐be catastrophic expense on cancer case management and the avoidable premature deaths due to late‐stage presentation of cancer cases. This is a way of working toward sustainability and community health empowerment in a resource‐limited setting. We aimed to examine the contribution of working with community organizations to increase access to cancer prevention and early detection services over 3 years period. We also sought to provide insight into the accrued benefit/monetary value of community assets and feasibility of conducting outreaches in community health centers, places of worships, schools, and workplaces in partnership with local communities.

## 2 METHODS

### 2.1 The outreach partnership model

As conceptualized in the ABCD model in Figure 2 as an ongoing community program, in outreaches, community partners mobilized the community, arranged for venues and other logistics including mass media (TVs and radio) airtime using their resources. UCI provided logistics such as equipment and supplies and multidisciplinary human resources to provide outreach services. Community health centers, faith‐based institutions, community‐based organizations (CBOs), community political leaders, and workplace managers were the key community‐based institutions and structures through which we provided cancer primary prevention and early detection services to the communities. We used mobile mammography van equipped with mammography and doctor/nurse space for clinical breast exam and mammography, with or without a recommendation for breast ultrasound and space for cervical screening. We used the digital rectal exam and PSA rapid tests combined with clinical criteria for prostate screening. Other cancer suspicious presentations and were clinically examined and referred for further investigation at the UCI.

### 2.2 Data source and cost comparison

We analyzed the quarterly UCI ‐CCCP reported output data without individual identifiers from July 2016 to June 2019 to compare the UCI ‐hospital‐based and outreach cancer awareness and screening services outputs as a short‐term assessment of the contribution of ABCD model‐based outreach. In cost comparison, the average unit cost for cancer screening was based on economic evaluation of cancer care done in various countries, notably; Cervical screening; Quentin et al,^9^ Cervical precancer treatment; Quentin et al,^9^ Breast screening, Gross et al.^10^ The average unit cost for managing early‐stage and late‐stage cancer management was based on; Cervical^7^; Ostensson et al (2015), Breast; Sun et al,^11^ Prostate cancer; localized, Fourcade et al^12^ and regional, De Oliveira et al.^13^, ^14^ The administrative overheads and procurement process related costs were not included in these considerations.

## 3 RESULTS

### 3.1 Community contribution

**FIGURE 3:**
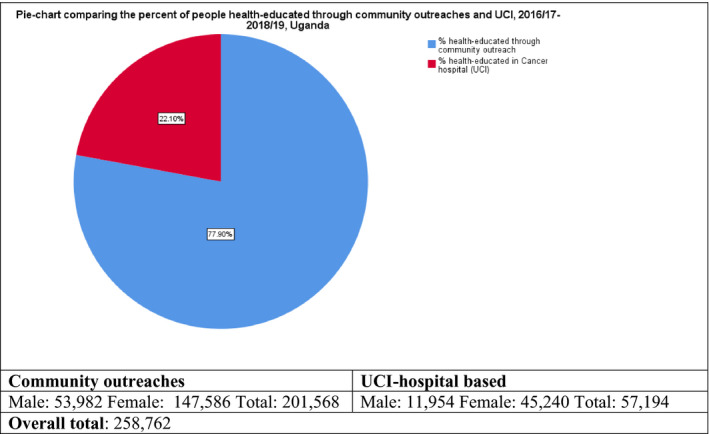
Proportion of people health‐educated through community partnered outreaches and UCI Hospital‐based sessions, 2016/17‐2018/19

**FIGURE 4:**
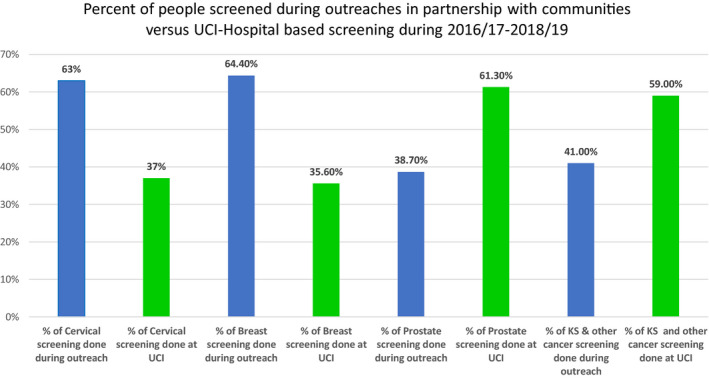
Proportion of people screened through community partnered outreaches and UCI Hospital‐based screening clinics, 2016/17‐2018/19

From July 2016 to June 2019, the community outreach model worked with 107 local partners either once or more than once to conduct 151 outreaches to extend cancer awareness and screening services in rural hard to reach and live populations in Uganda (Table 1). The number of outreaches and community partners worked with shows increasing trend over the three year. (Table 1). Of the total number of people health‐educated in group sessions and one‐on‐one on primary prevention of cancer, early detection and cancer management, 201 568 (77.9%) were reached through the outreach model (Figure 3 and Table 1). Ninety‐two (95%) of cancer awareness radio and TV talk‐shows conducted were sponsored by local partners (Table 1). Out of the total people screened; 22 795 (63.0%) cervical, 22 014 (64.4%) breast, and 4904 (38.7%) prostate screening clients benefited from the outreach model (Figure 4 and Table 2). The screen‐positive rates were higher in hospital‐based screening except for Prostate screening; cervical, 8.8%, breast, 8.4%, prostate, 7.1% than in outreaches; cervical, 3.2%, breast, 2.2%, and prostate, 8.2% (Table 2 and Table 3).

**TABLE 1:** Number of community partners, outreaches, and health education sessions, 2016/17‐2018/19, Uganda

Years	Number of community partners	Number of outreaches	Community sponsored TV & radio talks/interview	Govt sponsored TV & radio talks/interviews)	Total health educated	Health educated in outreach			Health educated at UCI		
						M	F	T	M	F	T
2018/19	42	61	22	1	143 436	30 289	87 563	117 852	5 461	20 123	25 584
2017/18	34	53	44	2	64 488	16 847	34 737	51 584	3 387	9 517	1 2904
2016/17	31	37	26	2	50 838	6 846	25 286	32 132	3 106	15 600	18 706
Total	107	151	92	5	258 762	53 982	147 586	201 568	11 954	45 240	57 194

**TABLE 2:** Comparing results of outreaches and UCI hospital‐based screening: Screened positive and cancer suspicious rates, 2016/17‐2018/19, Uganda

Year	Total screened in both outreaches & Uci	Cervical screening based on VIA or Pap‐smear					
		In outreaches with community support			UCI‐Hospital based screening clinics		
		Screened (% of total screened)	Positive (%)	Suspicious for cancer (%)	Screened (% of total screened)	Positive (%)	Suspicious for cancer (%)
2018/19	16 666	11 432 (67%)	457 (3.9%)	387 (3.4%)	5234 (33%)	271 (5.2%)	988 (18.9%)
2017/18	14 173	9059 (64%)	161 (1.7%)	179 (2.0%)	5114 (36%)	615 (12.0%)	1128 (22.0%)
2016/17	5490	2304 (42%)	115 (4.9%)	188 (8.2%)	3186 (58%)	309 (9.7%)	516 (16.2%)
Total	36 329	22 795 (63%)	733 (3.2%)	754 (3.3%)	13 534 (37%)	1195 (8.8%)	2632 (19.4%)
Breast screening bases on CBE or mammography with or without breast ultrasound scan							
Years	No. screened	In outreaches with community support			At UCI		
2018/19	16 186	11 067 (68.4%)	231 (2.1%)	221 (1.9%)	5119 (31.6%)	418 (8.2%)	317 (6.2%)
2017/18	11 774	8933 (75.9%)	164 (1.8)	216 (2.4%)	2841 (24.1%)	313 (11.0%)	179 (6.3%)
2016/17	6233	2014 (32.3%)	91 (4.5%)	134 (6.6%)	4219 (67.7)	296 (7.0%)	194 (4.6%)
Total	34 193	22 014 (64.4%)	486 (2.2%)	571 (2.6%)	12 179 (35.6%)	1027 (8.4%)	690 (5.7%)
Prostate screening based on DRE & PSA							
Years	Number screened	In outreaches with community support			UCI‐Hospital based screening clinics		
2018/19	8801	3143 (35.7%)	215 (6.85)	97 (3.1%)	5658 (64.3%)	186 (3.3%)	274 (4.8%)
2017/18	1692	856 (50.6%)	107 (12.5%)	95 (11.1)	836 (49.4%)	249 (29.7%)	216 (25.8%)
2016/17	2172	905 (41.6%)	82 (9.1%)	141 (15.5)	1267 (58.4%)	114 (8.9%)	191 (15.1%)
Total	12 665	4904 (38.7%)	404 (8.2%)	333 (6.8%)	7761 (61.3%)	549 (7.1)	681 (8.8%)

**TABLE 3:** Kaposi Sarcoma and other types of cancer^*^suspicious lesions based on clinical assessment, 2016/17‐2018/19, Uganda

Year	No. screened	Outreach in partnership with local communities						UCI‐Hospital based screening clinics					
		Clinical assessment			Suspicious for further investigation			Clinical assessment			Suspicious for further investigation		
		T	M	F	T	M	F	T	M	F	T	M	F
2018/19	1619	725 (44.8%)	341	384	101 (13.9%)	48 (47.5%)	53 (52.5%)	894 (55.2%)	367	527	172 (19.2%)	91 (53.0%)	81 (47.0%)
2017/18	1185	523 (44.1%)	317	206	66 (12.6%)	39 (59.0%)	27 (41.0%)	662 (55.9%)	373	289	136 (20.5%)	82 (60.0%)	54 (40.0%)
2016/17	1269	415 (32.7%)	201	214	45 (10.8%)	26 (57.7%)	19 (42.3%)	856 (67.3%)	359	497	72 (8.4%)	39 (54.2%)	33 (45.8%)
Total	4073	1663 (41.0%)	859	804	212 (12.7%)	113 **(53.3%)**	99 (46.7%)	2412 (59.0%)	1099	1313	380 (15.8%)	212 (55.8%)	168 (44.2%)

^*^Excluding breast, cervical and prosate cancer suspicous lesions

### 3.2 Comparing cost of screening and managing cancer from precancer to advanced cancer

The unit cost incurred in Cancer screening was thousands‐folds less than the unit cost of managing in any stage of cancer disease (Table 4). The cost of managing cancer increases with the stage of cancer progression. Regarding Cervical cancer; the average unit cost for managing localized Cervical cancer cases was 2941 times (18 000/6.12) higher than the average unit cost of cervical screening. The average unit cost for managing advanced cervical cancer (Regional or distant) was 6005 times (36 751.07/6.12) higher than the average unit cost of screening. The average unit cost for managing advanced Cervical cancer (Regional or distant) was 1314 times (36 751.07/27.96) higher than the average unit cost of precancer treatment of Cervical lesions using cryotherapy. The average unit cost of managing advanced Cervical cancer cases was two‐folds (36 751.07/18 000) higher the unit cost for managing localized Cervical cancer cases.

**TABLE 4:** Comparing the unit costs for screening, precancer treatment, managing localized, and advanced cancer

Cancer type screened	No. Screened (a)	Average unit cost of screening per client US$ (b)	Average direct screening total cost incurred US$ (c)	No. of precancers/screen positives (d)	Average unit cost of treating precancerUS$ (e)	Cost incurred in treating precancers US$ (f)	Total cost of screening US$ (g) = c + f	Suspicious cancer lesions (i)	Est.Min. No. that would be detected with cancer(j) d + i	Early stage cancer = 20% of j	Late stage cancer = 80% of j	Est.Unit cost for localized cancer mgt	The would be cost incurred in managing localized cancer US$	Est. Unit cost for managing advanced cancer (Regional or distant) US$ (k)	The would be cost incurred in managing regional & distant cancer US$ (l)	The would‐be total cost of managing cancer disease
Cervical	36 329	6.12^a^	2 223 334	1928	27.96^a^	53 907	2 277 241	3386	5314	1063	4251	18 000^b^	19 134 000	36 751.07^b^	156 228 799	175 362 799
Breast	34 193	42^a^	1 436 106	1513	3000^a^	4 539 000	5 975 106	1261	2774	555	2219	29 724^b^	16 496 820	62 108^b^	137 817 652	154 314 472
Prostate	12 665	30^a^	379 950	953	4286	4 084 558	4 464 508	1014	1967	393	1574	6369^b^	2 503 017	18 000^b^	28 332 000	30 835 017
Total	83 187		4 039 390	4394		8 677 465	12 716 855	5661	10 055	2011	8044		38 133 837		322 378 451	360 512 288

^a^ The average unit cost for Cancer screening were based on; cervical screening, Quentin et al,^9^ Cervical precancer treatment, Quentin et al,^9^ breast screening, Gross et al^10^ while Prostate screening was based on average market cost.

^b^ The average unit cost for managing early stage and late stage cancer were based on; Cervical, Ostensson et al,^7^ Breast, Sun et al^11^ and Prostate cancer; localized prostate cancer, Fourcade et al;^12^ regional prostate cancer, De Oliveira et al.^13^, ^14^

In Breast cancer scenario; the average unit cost for managing localized Breast cancer cases was 708 times (29 724/42) higher than the average unit cost of screening. The average unit cost for managing advanced Breast cancer (Regional or distant) was 1479 times (62 108/42) higher than the average unit cost of breast screening. The average unit cost for managing advanced Breast cancer was 21 times (62 108/3000) higher than the average unit cost of precancer treatment of Breast cancer lesions using Lumpectomy. The average unit cost for managing advanced Breast cancer (Regional or distant) was two times (62 108/29 724) times higher than the average unit cost for managing localized cervical cancer cases.

In the context of prostate cancer; the average unit cost for managing localized Prostate cancer cases was 212 times (6369/30) higher than the average unit cost of screening. The average unit cost for managing advanced Prostate cancer (Regional or distant) is 600 times (18 000/30) higher than the average unit cost of screening. The average unit cost for managing advanced Prostate cancer (Regional or distant) was 4.2 times (18 000/4286) higher than the average unit cost of precancer treatment of Prostate lesions using surgical intervention. The average unit cost for managing advanced Prostate cancer (Regional or distant) was 2.8 times (18 000/6369) higher than the average unit cost for managing localized cervical cancer cases.

## 4 DISCUSSION

### 4.1 The contribution of working with communities as partners to leverage health resources

In regard to cancer awareness efforts through health education and use of mass media channels on primary prevention and early detection of cancer, more people were reached through community partnership. For instance; 77.9% of people were health‐ educated through outreaches and ninety‐two (95%) cancer awareness TVs and radio talk‐shows were sponsored by local community partners.

Pertaining to cancer screening, overall, more people were screened in outreaches except for prostate screening; 63.0% cervical, 64.4% breast and 38.7% prostate screening. This is probably due to long‐distance and associated cost involved in travelling to a tertiary hospital for cancer information and screening services. Therefore, bringing services closer to the people especially those in rural hard to reach and hard to live areas is an opportunity for the community members.

It has been shown in some countries that a well‐organized outreach model could bridge the cancer health‐equity disparity especially for the rural residents and socio‐economically disadvantaged individuals. Countries like South Africa, Nigeria, Canada, and the USA used mobile cancer‐preventive outreaches to increase access to primary prevention and early detection services.^29^, ^30^, ^31^ Therefore, replication of such model is crucial especially in low‐come countries and territories with inadequate number of cancer detection centers and hospitals.

Health promotion settings such as communities, schools, workplaces, places of worships such as churches and mosques are places and social context where people engage in daily activities in which environmental, organizational, and personal factors interact to affect health and well‐being, where people actively use and shape the environment and thus create or solve problems relating to health. Therefore, the ABCD model can be conceptualized for application in all these health promotion settings. For instance, in England, a review of ABCD model in school health promotion setting found that ABCD approach was effective and concluded that engaging school communities through ABCD model was a promising practice.^32^ The principles of ABCD model have also been shown to improve the health of people with chronic conditions in community health nursing interventions.^33^

In our community outreach model, it was also observed that the screen‐positive rate and cancer suspicious rate were higher in hospital‐based screening than in outreaches. This could be so because some people might choose to visit the hospital only when they evaluate themselves to be most at risk or are driven by early warning signs and symptoms. Therefore, cancer awareness and screening outreaches, especially in rural areas, could increase access to cancer awareness and early detection.

This demonstrates the benefit and feasibility of working with communities as local partners especially in primary prevention and early detection of cancer. In communities, places such as community health centers, schools, places of worships, and workplaces are organized avenues for partnership and community‐based assets. Community primary health‐care facilities, faith‐based institutions, community‐based organizations (CBOs), community leaders, and workplace managers are resourceful community‐based institutions and structures through which cancer primary prevention and early detection services to the communities could be structured.

From the annual UCI’s work plan, the average annualized budget for Cancer Outreach Service during 2016/17, 2017/18 and 2018/19 was 188 775 000 Uganda shillings (54 000 USD), equivalent to 162 000 USD spread over the three financial years. This excludes the costs of training health workers and developing cancer information, education, and communication materials that were funded through the African development bank “East Africa Centre of Excellence for Oncology project” at the UCI. When overhead cost including salaries except allowance for outreach staff facilitation is not considered, the 162 000 USD cancer outreach budget for the three financial years is only 1.3% of the total 12 716 855USD estimated cost of cancer screening during the three years period. Meaning that greatest portion (98.7%) of the outreach cost was absorbed by through community partnership. This portion of 98.7% was covered by the monetary and nonmonetary community assets like arrangement of venues, local community health facilities, and social mobilization among others. The UCI’s government funding was used in screening supplies and staff allowances.

These illustrate the benefit of working in collaboration with communities as partners and outreaching the rural population. Most importantly it is the cost‐sharing, leverage for scarce resources and increased capacity to sustain programs that promote primary prevention and early detection of cancer. Knowing the fact that low‐income settings are characterized by inadequate investment in national cancer control, the contribution of locally available community assets, whatever small it is, should not be ignored.

### 4.2 Comparing cost of screening and managing cancer from precancer to advanced cancer

#### 4.2.1 A snapshot into the costing of cancer screening and treatment

The two common approaches applied in costing health‐care services are; “incremental” and “base case” scenarios.^34^ For simplicity, incremental economic costs involve two major steps; (1) the ingredients approach, that is quantities of resources used and (2) unit costs or prices are assigned to resources consumed.^34^, ^35^ Alternatively, the “Base‐case scenario” is applied. In the “Base‐ case scenario” the assumed resources used per client or patient is multiplied by their estimated unit costs and the influence of alternative assumptions for input parameters is tested through sensitivity analyses.^34^, ^35^ The “Base‐case scenario” make assumptions for the costs of inputs, number of clients screened and treated by each service provider, effective working time of capital and staff, costs of training, duration of screening or patient management per client.^34^, ^35^

#### 4.2.2 Screening and treatment cost

The cost of screening, diagnosis and treatment of the three commonest Cancers in Uganda; Cervical, Breast, and Prostate cancer vary significantly even if they were of the same disease‐stage. When you compare the cost of cancer awareness and screening and cost of managing cancer (diagnosis, staging, treatment, and follow‐up), the unit cost incurred in cancer screening is thousand‐fold less than the unit cost of managing any stage of cancer. It has been shown that the cost of managing cancer increases with the stage of cancer progression.^7^ For example, in this study, the average unit cost for managing localized Cervical cancer cases is 2941 times higher than the average unit cost of cervical screening. The average unit cost for managing advanced cervical cancer (Regional or distant) is 6005 times higher than the average unit cost of screening. The average unit cost for managing advanced Cervical cancer (Regional or distant) is 1314 times higher than the average unit cost of precancer treatment of Cervical lesions using cryotherapy. The average unit cost of managing advanced Cervical cancer cases is two‐fold higher the unit cost for managing localized Cervical cancer cases. This means residents of low‐income countries are likely to continue experiencing catastrophic expenditure for cancer treatment if no local options of community assets are tapped into primary prevention and early detection of cancer.

Similar catastrophic costs are observed in other types of cancer. For instance, the average unit cost for managing localized Breast cancer cases is 708 times higher than the average unit cost of screening. The average unit cost for managing advanced Breast cancer (Regional or distant) is 1479 times higher than the average unit cost of breast screening. The average unit cost for managing advanced Breast cancer is 21 times higher than the average unit cost of precancer treatment of Breast cancer lesions using Lumpectomy. The average unit cost for managing advanced Breast cancer (Regional or distant) is two times higher than the average unit cost for managing localized cervical cancer cases. In the context of prostate cancer; the average unit cost for managing localized Prostate cancer cases is 212 times higher than the average unit cost of screening. The average unit cost for managing advanced Prostate cancer (Regional or distant) is 600 times higher than the average unit cost of screening. The average unit cost for managing advanced Prostate cancer (Regional or distant) is 4.2 times higher than the average unit cost of precancer treatment of Prostate lesions using surgical intervention. The average unit cost for managing advanced Prostate cancer (Regional or distant) is 2.8 times higher than the average unit cost for managing localized cervical cancer cases.

Therefore, the ABCD model recognizes the power of local community members, associations, the supportive functions of both public and private institutions and bring it to bare in facilitating sustainable community health and development interventions.^36^, ^37^, ^38^ Therefore, instead of only profiling the health needs of a community, we need also to look at the assets in the community, mobilize these assets and capacities to leverage resources for cancer control services. This is not a “new” approach to public health; however, it recognizes the important role of communities in public health practice.^39^ Rita Agdal et al in a qualitative meta‐synthesis found that ABCD driven community health projects targeting children and youths had the highest level of participation in health promotion interventions.^40^

This re‐affirms that prevention of cancer is the best option to invest in and if cancer develops in an individual, it should be detected early enough when treatment outcome and cost are favorable to the individual and the government. Therefore, deliberate efforts are required to outreach such communities especially rural, hard‐to‐reach, and hard‐to‐live populations with affordable or cost‐shared cancer prevention and early detection services. Community‐based resources could mitigate part of the cost involved in cancer prevention and early‐detection services. This is because rural and socioeconomically disadvantaged populations experience the worst difficulties in accessing cancer prevention and early detection services in many low‐income countries. However, the best option is an equitable comprehensive national cancer control program.

## 5 CONCLUSION

Working in collaboration with communities and community‐based organizations through assets‐based community development model is feasible. This helps in cost‐sharing, leverage for scarce resources and it is an ecological approach to bridging cancer health disparities. In low‐ and middle‐income countries with limited number of cancer hospitals and early detection centers, increasing population cancer awareness to promote prevention and early detection should leverage from community‐based assets through an organized outreach model as an intermediate solution. Deliberate efforts are required to outreach communities in low‐income settings with affordable cancer preventive and early detection services. However, an equitable and cost‐effective comprehensive national cancer control program is a prerequisite.

## CONFLICT OF INTEREST

The authors have declared that no competing interests exist.

## AUTHORS' CONTRIBUTIONS

Alfred Jatho: Community partnership concept, outreach coordination, community mobilization, mass media (TVs and Radio talk shows) cancer awareness, health education sessions, activity reports, manuscript drafting. Noleb Mugume Mugisha: Mass media (TVs and Radio talk shows) cancer awareness, screening, activity reports, and manuscript review. James Kafeero: mass media (TVs and Radio talk shows) cancer awareness, screening, and manuscript review. George Holoya: Screening and manuscript review. Fred Okuku: Outreach concept, design, and manuscript review. Nixon Niyonzima: Training and manuscript review.

## ETHICAL STATEMENT

Not applicable.

## CONSENT FOR PUBLICATION

Not applicable.

## Data Availability

All relevant data are within this report.
